# Checkpoint inhibitors in endometrial cancer: preclinical rationale and clinical activity

**DOI:** 10.18632/oncotarget.20042

**Published:** 2017-08-08

**Authors:** Gloria Mittica, Eleonora Ghisoni, Gaia Giannone, Massimo Aglietta, Sofia Genta, Giorgio Valabrega

**Affiliations:** ^1^ Department of Oncology, University of Turin, Turin, Italy; ^2^ Division of Medical Oncology-1, Candiolo Cancer Institute-FPO- IRCCS, Candiolo, Italy

**Keywords:** endometrial cancer, immunotherapy, tumor infiltrating lymphocytes (TILs), polymerase epsilon (POLE)-ultra-mutated, microsatellite instability (MSI)

## Abstract

**Context:**

Treatment of advanced and recurrent endometrial cancer (EC) is still an unmet need for oncologists and gynecologic oncologists. The Cancer Genome Atlas Research Network (TCGA) recently provided a new genomic classification, dividing EC in four subgroups. Two types of EC, the polymerase epsilon (POLE)-ultra-mutated and the microsatellite instability-hyper-mutated (MSI-H), are characterized by a high mutation rate providing the rationale for a potential activity of checkpoint inhibitors.

**Materials and Methods:**

We analyzed all available evidence supporting the role of tumor microenvironment (TME) in EC development and the therapeutic implications offered by immune checkpoint inhibitors in this setting. We performed a review on Pubmed with Mesh keywords ‘endometrial cancer’ and the name of each checkpoint inhibitor discussed in the article. The same search was operated on clinicaltrial.gov to identify ongoing clinical trials exploring PD-1/PD-L1 and CTLA-4 axis in EC, particularly focusing on POLE-ultra-muted and MSI-H cancer types.

**Results:**

POLE-ultra-mutated and MSI-H ECs showed an active TME expressing high number of neo-antigens and an elevated amount of tumor infiltrating lymphocytes (TILs). Preliminary results from a phase-1 clinical trial (KEYNOTE-028) demonstrated antitumor activity of Pembrolizumab in EC. Moreover, both Pembrolizumab and Nivolumab reported durable clinical responses in POLE-ultra-mutated patients.

**Conclusions:**

Immune checkpoint inhibitors are an attractive option in POLE-ultra-mutated and MSI-H ECs. Future investigations in these subgroups include combinations of checkpoints inhibitors with chemotherapy and small tyrosine kinase inhibitors (TKIs) to enhance a more robust intra-tumoral immune response.

## INTRODUCTION

Endometrial Cancer (EC) is expected to be the 4th most common malignancy among women and the 6th leading cause of death in 2017 [1]. EC is frequently associated with Lynch Syndrome (LS). also called hereditary non-polyposis colon cancer (HNPCC), [2], an autosomal dominant genetic disorder which confers an increased risk of developing different kind of tumours, [3].

LS , is characterized by alterations in genes involved in DNA mismatch repair (MMR), such as MLH1, MSH2, MSH6, PMS2 and EPCAM, resulting in microsatellite instability (MSI) [4]. For the 67% of EC patients diagnosed at an early stage, 5-year overall survival is of 95% after surgery with or without radiotherapy. Instead EC patients diagnosed at a late stage have a 5-year survival rate of only 17% [1, 5]; these patients are candidate to systemic treatment with palliative intent, including chemotherapy, among which carboplatin-paclitaxel doublet is the most effective scheme [1], and endocrine treatments [6]. Up to date there is no standard second line therapy [7].

In this review, we will concentrate on the scientific background supporting the clinical development of immune checkpoint inhibitors in advanced and recurrent disease with a specific focus on the role of tumor microenvironment (TME).

### The “modern” molecular classification: beyond Bokhman's dual scheme

EC is an heterogeneous disease with various histological subtypes, which have different pathogenesis, prognosis and sensitivity to different therapeutic agents [8].

In the past decades, EC has been classified in two subtypes, respectively named Type I and Type II according to Bokhman's model [9], based on clinical characteristics integrated with histological features and hormone receptor (HR) status. Type I is the most common EC (60–70% of cases); it includes grade1 and 2 endometrioid cancer with a high presence of Estrogen Receptors (ERs) and Progesteron Receptors (PgRs). It is related to increased Estrogen levels and endometrial hyperplasia and it is usually associated with a good prognosis (median 5-year survival rates of 85.6%). The most frequently altered pathway in Type I is PTEN-PIK3/AKT/mTOR (PTEN is mutated in approximately 52–78% of lesions), followed by KRAS mutations (15–43%), ARID1A and Β-catenin alterations [10]. MSI is present in one third of type I EC [11].

Type II EC comprises Grade 3 endometrioid, serous or clear cell HR negative cancers, and it is usually associated with endometrial atrophy [12]. TP53 is the hallmark alteration of this subtype. Patients with type II EC generally show an advanced stage at diagnosis, a low response rate to therapies and a poor prognosis [11, 12].

This dualistic model has recently been expanded in consideration of new knowledge concerning genomic and transcriptomic analysis [13, 14]. In 2013 TGCA (The Cancer Genome Atlas Research Network) [14] published the first genomic characterisation of EC. Results of this study allowed EC classification in four different subtypes, based on somatic mutations, copy number alterations and microsatellite instability:

POLE-ultra-mutated malignancies, representing 6.4% of low-grade and 17.4% of high-grade endometrioid tumours, are characterized by a high mutation rate (232 × 10^–6^ mutations/Mb); their hallmarks are somatic mutations in the exonuclease domain of POLE that encodes the catalytic subunit of DNA polymerase epsilon. Loss of function of this polymerase, which plays a relevant role in DNA repair, leads to a high frequency of C>A transversions, few copy number alteration and microsatellite stability (MSS). PTEN, PIK3R1, PIK3CA, RAS are frequently mutated [12, 14, 15]. Despite the histological grade, this group is associated with good prognosis [16–20].

MSI-hyper-mutated (MSI-H) tumors represent 28.6% of low grade and 54.3% of high-grade endometrioid EC [14, 15, 21]. They show MSI and high mutation rate (18 × 10^−6^ mutations/Mb) related to defects in MMR system (the most implicated genes are MLH1, MSH2, MSH6, PMS2), both in sporadic and hereditary EC. PTEN mutations and subsequent alterations of the PTEN-PIK3CA pathway recur in this subgroup [22]. Further genetic abnormalities are frequent, like RPL22 frameshift deletions and KRAS mutation. There is no significant correlation between MSI and outcome in ECs patients [23].

Copy-number low EC is characterized by a low mutation rate (2.9 × 10^–6^ mutations/Mb) and MSS. It is frequently a low-grade endometrioid cancer (in TGCA 60%of low-grade and only 8.7% of high grade EC were MSS copy low); PTEN and PIK3CA are mutated in 77% and 53% of cases respectively [14, 15]. Other common alterations involve WNT-B catenin axis; RAS mutation is rare; PgR levels are high and this finding predicts usefulness of endocrine therapy [21, 24]. Prognosis is similar to MSI-H tumors without a clear correlation between this subtype and clinical outcome.

Copy-number high serous like subgroup includes mainly serous and mixed histology tumors with some high grade endometrioid EC. It has a low mutation rate (2.3 × 10^−6^ mutations/Mb) and a small load of copy number aberrations. TP53 is commonly mutated (92%), whereas KRAS and PTEN mutation are infrequent; 25% of the serous-like tumours are ERBB2-amplified [14, 21]. Prognosis of these patients is poor [12, 15].

**Figure 1:**
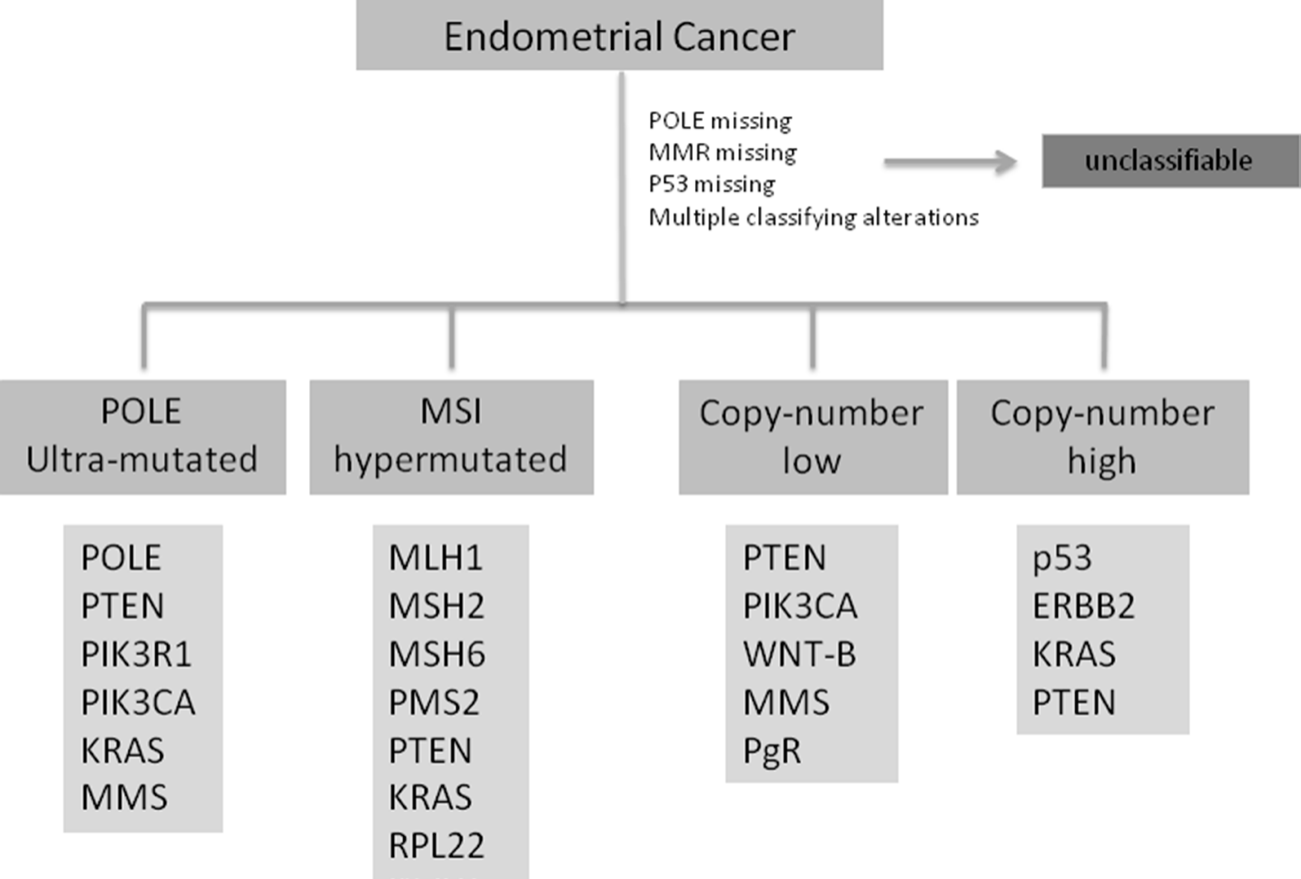
Shows the ECs classification according to TGCA including the most common genetic alteration in each subtype POLE: polymerase epsilon; MMR:mismatch repair; p53: tumor protein p53; PTEN: phosphatase and tensin homolog; PIK3: phosphatidylinositol-4,5-bisphosphate 3 kinase; KRAS: Kirsten rat sarcoma viral oncogene homolog; MMS: microsatellite stability; MSI: microsatellite instability; MLH: mutL homolog 1; MSH2: mutS homolg 2; MSH6: mutS homolog 6; RPL22: 60S ribosomal protein L22; ERBB2: human receptor tyrosine-protein kinase erbB-2; WNT-B: WNT-beta catenin pathway; PgR: progesteron receptor.

This new classification, reported in Figure 1, could be comparable to the already well known pathogenesis model of colon-rectal cancer [25] and may represent a step forward in defining prognosis of EC patients and may help in improving clinical trial design with targeted agents [24, 26, 27].

As recently reported, normal endometrium has a peculiar immune system; indeed, it has a dualistic role: it should be active against sexual pathogens and should allow the growth of an allogenic and “non-self” fetus [28, 29]. This behavior is regulated by sex hormones that influence therefore the TME, especially defining the typology of adaptive immune cells [30].

It is well known that immune cells can recognize and eliminate cancer cells through the identification of tumor –specific antigens (TSA) and tumor-associated antigens (TAA) [31].

**Figure 2:**
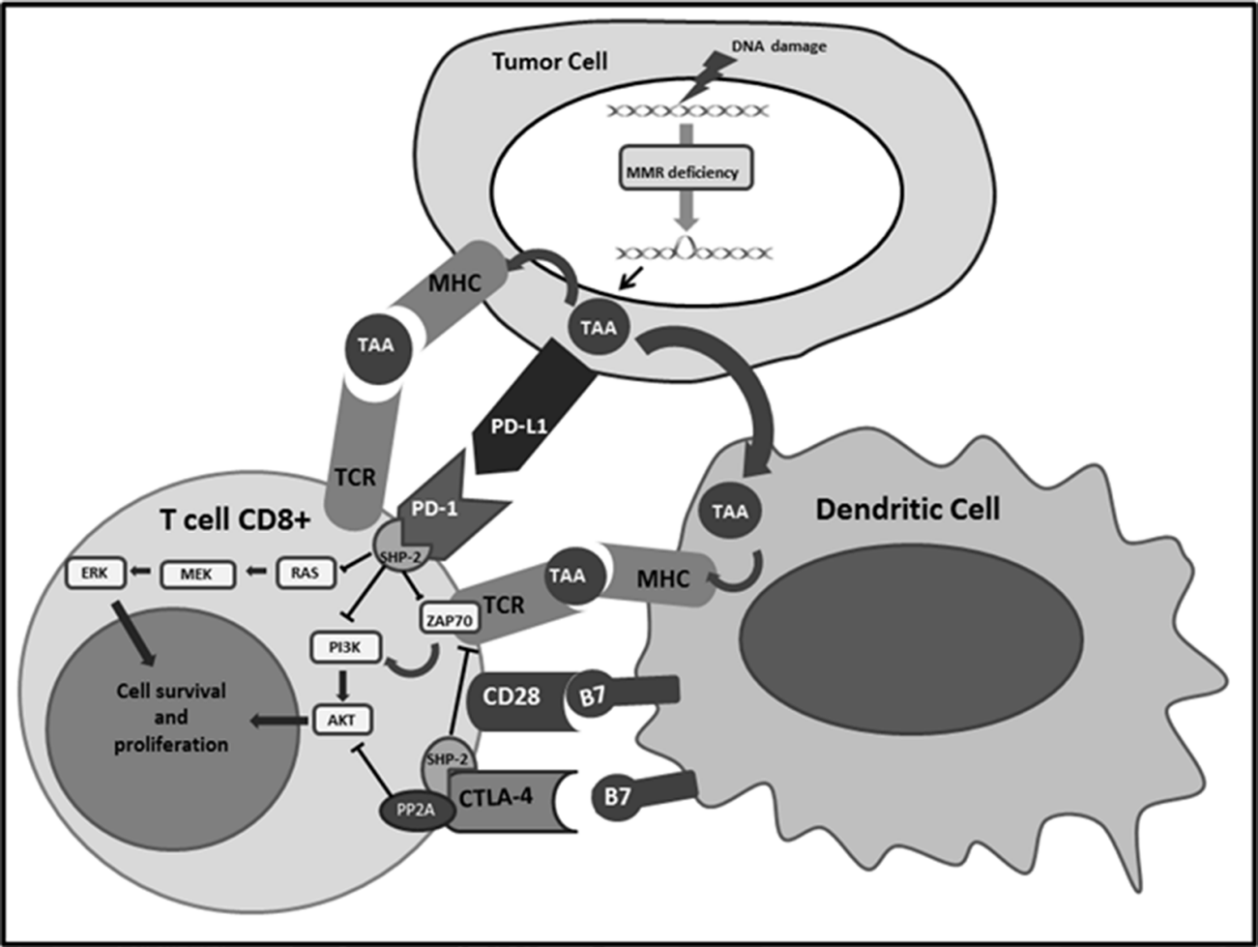
Shows the interactions of PD-1 and CTLA-4 expressed on the surface of the T cells with the respective ligands and the subsequent activation of immune checkpoint signalling pathways that inhibit lymphocytes survival and proliferation CTLA-4:Cytotoxic T-Lymphocyte Antigen 4; MHC: Major histocompatibility complex; MMR: Mismatch repair PD-1: Programmed cell death protein 1; PD-L1: Programmed death-ligand 1; TAA: Tumor associated antigen ;TCR: T-cell receptor.

Physiologically, when TAA are recognized by T cells they are handled, converted into small fragments and finally presented by antigen-presenting cells (APCs) after loading on major histocompatibility complex (MHC) class I and II. Usually, immune response activation is elicited if two positive signals are present. The first one is the interaction between MHC molecules and T cell receptors (TCR); the second one is the connection of the co-stimulatory receptor CD28, present on T cells’ surface, with its ligand B7 on APCs. In order to avoid autoimmune reaction CD28 has a competitor for binding B7, the cytotoxic T lymphocyte antigen-4 (CTLA-4), which carries an inhibitory signal. This negative feedback is mostly represented within secondary lymphoid organs, while the inhibitory pathway more frequently present within peripheral TME is the connection between the programmed cell death-1 (PD-1) receptor on the T cells, and the programmed cell death ligand-1 and 2 (PD-L1 and PD-L2) on the tumor cells surface [32, 33]. Different molecular patterns are involved downstream this interaction, such as inhibition of PI3K/AKT and Ras/MEK/Erk pathways, through down-regulation of PTEN and PLC-γ1 respectively [34–36] (Figure 2). Inflammatory cytokines, as interferon, IL-4 and IL-10, generated after recognition of TAA and TSA stimulates PD-1 and PD-L1 over-expression, lead to down-regulation of T-cell reaction and create the mechanism called “adaptive immune resistance” [37]. Other immune checkpoints seem to play a role in adaptive immune resistance, such as Lymphocyte Activation Gene 3 (LAG-3) and indoleamine 2,3-dioxygenase (IDO), both up-regulated in POLE and MSI-H subtypes [38, 39]. Among gynecological cancer, EC show the highest expression of PD-1 and PD-L1,75 % and 25–100% respectively [40]. Moreover, Vanderstraeten and coll. analyzed other immune-related molecules and reported that B7-H4, responsible of another inhibitory pathway of CD4^+^ and CD8^+^ T cells, is present in 90% of EC specimens, while IDO is expressed only in 21% of EC samples [39]. These findings confirm an important role of PD-1/PD-L1 pathway and suggest B7-H4 signal as a potential new therapeutic target.

The correlation between PD-L1 expression and patient's outcome is controversial, since has been associated with a worse prognosis in some tumors, like non-small cell lung cancer (NSCLC) [41], kidney [42–44] and bladder [45] cancer, and with a good one in melanoma [46]. Currently, PD-L1 is routinely analyzed in advanced NSCLC in order to prescribe checkpoint inhibitors, even if is still controversial which is the best cut-off to define positivity and which the best antibody to detect the expression on immunohistochemistry (IHC) assay [47]. PD-L1 detection is regularly used also in the treatment of kidney and bladder cancers [48].

**Figure 3:**
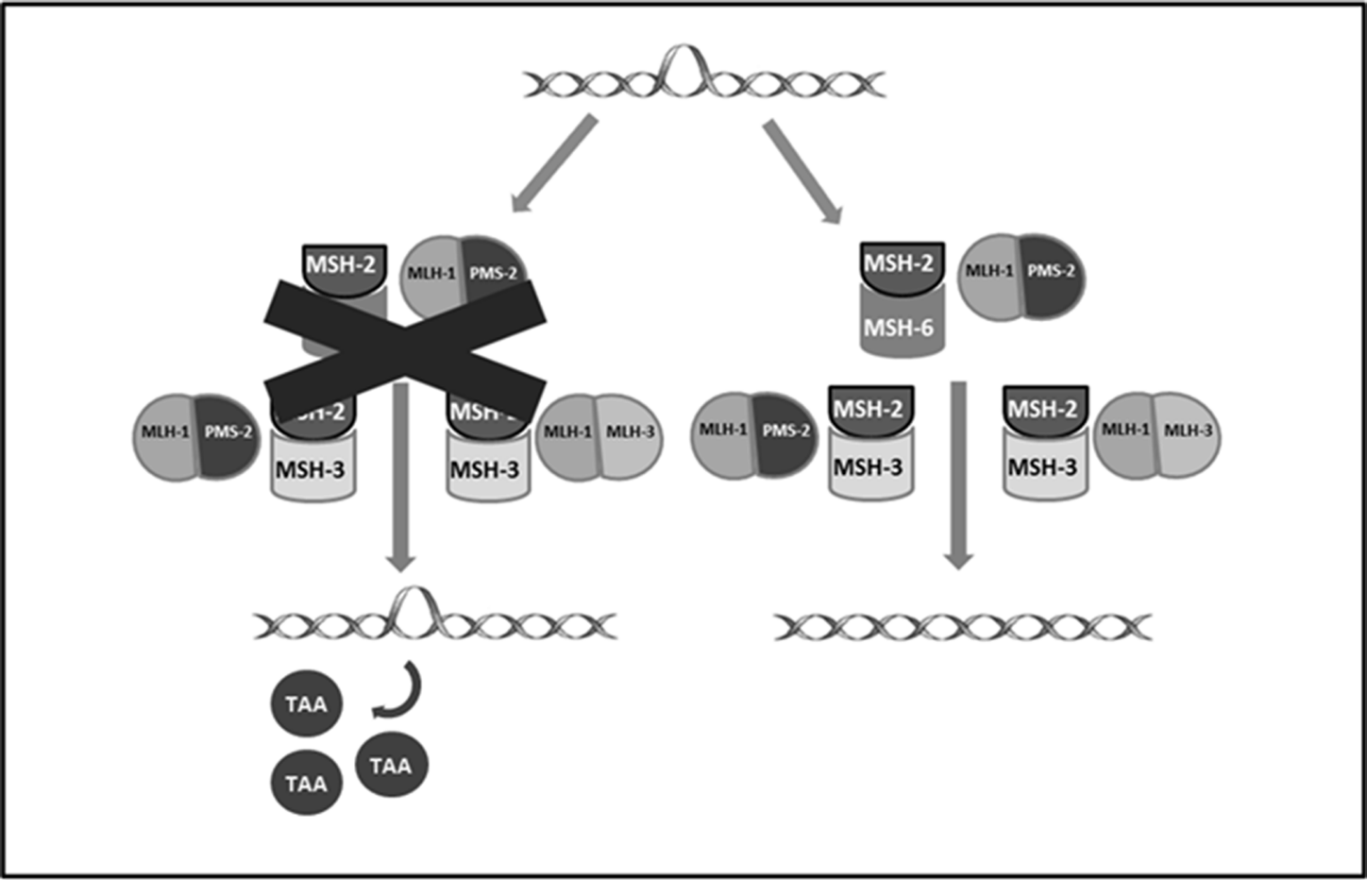
Shows proteins involved in DNA mismatch repair system and the formation of neoantigens resulting from their deficiency TAA: tumor associated antigen.

The prognostic value of the expression of this inhibitory pathway, as the role of other components of tumor microenvironment (TME), such as tumor infiltrating lymphocytes (TILs), is currently under investigation in EC. PD-1 and PD-L1 are more frequently reported in POLE-mutated and MSI-H tumors. This pathway might account for more aggressive histopathologic features observed in POLE-mutated, as reported above, even if these tumors have a good prognosis related to a higher number of CD3^+^ and CD8^+^ TILs that prevent disease dissemination [49, 50]. POLE-mutated and MSI tumors have an active TME not only for the high number of TILs, but also for the huge amount of tumor specific neo-antigens, generated by genetic alteration acquired due to impaired DNA replication fidelity (POLE) and defective DNA MMR system (MSI-H) [4, 14] (Figure 3). Recent studies have characterized the different cell populations constituting TME. The presence of TILs appears associated with a better outcome in many different kinds of cancers such as melanoma [51], esophageal [52], breast [53], colorectal [54] and ovarian cancer [55, 56]. In EC, in 2009 de Jong and colleagues assessed the number of CD8^+^ (cytotoxic T-lymphocytes, CTL), FOXP3^+^ (regulatory T-lymphocytes, Treg) and CD45R0^+^ (memory T-lymphocytes) TILs by IHCon tissue microarrays [57]. High numbers of CTL and a high CD8^+^/ FOXP3^+^ ratio were correlated with a longer disease free survival (DFS), while high levels of CTL and presence of CD45R0^+^ memory cells were associated with a greater overall survival (OS). In the multivariate analyses high presence of CTL was an independent prognostic factor for longer OS in the entire EC population (HR 0.48, *p* = 0.019), with a major impact in type II EC (HR 0.17, *p* < 0.001), whereas high CD8^+^/ FOXP3^+^ ratio is the factor independently correlated with prolonged survival in type I cancers. The prognostic role of CD8^+^/ FOXP3^+^ ratio was confirmed also by subsequent investigations [58, 59]. The studies focused on Treg alone reported a correlation with tumor stage, grade and myometrial invasion but not with survival [60, 61]. Recently, Pakish and colleagues evaluated the EC TME matching and comparing MSI-H with MSS (POLE-mutant cases and cases with unknown POLE status were excluded) [4]. They reported an increased number of immune cells in specimens from MSI-H EC including granzyme B+ cells, activated CTL and PD-L1 + cells. The authors also compared sporadic MSI-H EC with those related to LS (LS MSI-H): they observed an increased level of CD8+ cells and activated CTL with a lower number of macrophages in stroma of LS MSI-H EC while sporadic MSI-H EC showed a higher level of PD-L1 + macrophages. The analyses performed by the TransPORTEC consortium on 116 high-risk ECs, published also in 2017, confirmed that POLE-mutant and MSI-H tumors are characterized by higher numbers of tumor-infiltrating T cells. These two subgroups are both neoantigen-rich and with a huge density of PD-1 and PD-L1 expression and so are the perfect candidates for immune checkpoint inhibitors, as further reported [62]. In order to avoid the activation of the inhibitory pathways described above different antibodies have been developed, targeting PD-1, PD-L1, PD-L2 and CTLA-4 These molecules, known as “checkpoint inhibitors”, exhibited efficacy and durable clinical response in various cancer types and have already been approved for NSCLC [63, 64], melanoma [46, 65–69], kidney [70], bladder [71, 72] and Hodgkin Lymphoma [73, 74].

### Clinical activity of checkpoint inhibitors in endometrial cancer

The first evidence for clinical activity of immunotherapy in EC derive from a phase II trial published in 2015 by Le and colleagues which enrolled 41 patients [75]. Study population was divided in three cohorts, including respectively patients with MMR-deficient colorectal cancer, patients with MMR proficient colorectal cancer and patients with MMR-deficient cancers other than colorectal cancer; in third cohort were also included two patients affected by EC. All patients were treated with the anti-PD-1 Pembrolizumab. Authors reported a higher immune-related objective response rate (ORR) and 20-week immune related progression free survival (PFS), 40% and 78%, respectively, in the MMR deficiency cohorts, versus 0% and 11% in MMR proficient colorectal patients. In cohort C, including the 2 EC patients, immune-related ORR and PFS were 71% and 67%, respectively. This is a pivotal study reporting for the first time a connection between TME, genotype and response to checkpoint inhibitors, a significant step forward in the identification of predictors of response, as discussed in the next section.

Recently, Ott and colleagues published the results of KEYNOTE-028 trial, a phase Ib study involving 24 patients with advanced EC [76]. All patients were treated with Pembrolizumab 10 mg/kg every two weeks for up to 24 months or until confirmed progression, intolerable toxicity, death, or consent withdrawal. Overall Response rate observed was 13%. Three patients obtained a partial response and other three achieved a stable disease. Authors reported a six-months PFS and OS rates of 19.0% and 68.8% respectively. Drug-related adverse events occurred in 54.2% of patients; most common were pruritus, asthenia, fatigue, pyrexia, and decreased appetite. No patients died or discontinued Pembrolizumab because of toxicities. Interestingly, Ganesan and collegues reported the case of a durable partial response with Pembrolizumab in one patient with POLE-mutation [77]. Pembrolizumab has been tested in metastatic EC also in combination with Lenvatinib, a multikinase-inhibitor with antiangiogenic activity. Makker and colleagues recently presented the results of a phase I / II trial in which 23 patients received Lenvatinib 20 mg/day and Pembrolizumab 200 mg every three weeks. The authors reported an ORR of 48% and a DCR of 96%. The most common adverse events were hypertension, fatigue, arthralgia, diarrhea and nausea [78].

In 2016 Santin and colleagues reported the cases of two patients with recurrent EC refractory to surgery, chemotherapy and radiotherapy treated with the anti-PD-1 Nivolumab [79]. The two women were respectively affected by a mixed clear cell and endometrioid POLE mutated EC and by a serous MSH6 mutated EC. Both patients were treated with Nivolumab 3 mg/kg biweekly. CTL infiltration and PD-L1 expression were evaluated on a pretreatment biopsy. The first patient showed a moderate amount of peri and intratumoral lymphocytic infiltrate. Moreover, a weak membranous PD-L1 expression was reported in about 5% of the tumor cells, whereas the peri- and intratumoral lymphocytes were PD-L1 negative. In the second patient the peri-tumoral lymphocytic infiltrate was moderate, whereas there were fewer CD8-positive lymphocytes within the tumor cell nests. PD-L1 was observed in approximately20% of peri- and intratumoral lymphocytes, while no significant PD-L1 expression was observed for cancer cells. In this case authors evaluated also p53 expression by immune histochemistry which revealed a wild type pattern. Both patients obtained a persistent clinical response to Nivolumab confirmed by a CT scan respectively at 7 and 9 months from the start of immunotherapy. No severe toxicities were reported.

Antibodies against PD-L1 were also tested for the treatment of endometrial carcinoma.

Fleming and colleagues reported the results of a phase Ia study in which 15 women with EC received Atezolizumab 15 mg/m 2 every three weeks. The majority of patients were MSS (7/15) or MSI unknown ( 7/1), only one patient has a MSI-H disease. Authors also evaluated the status of PD-L1: 33% of patients had an expression of PD-L1 greater than 5% on immune cells while in the remaining 67% the PD-L1 expression was lower. Two patients obtained a partial response and other two achieved a stable disease with an ORR of 13% and a DCR of 27%. Both responders had an expression of PD-L1 greater than 5 %, one had MSS disease heavily infiltrated with TILs, the other had a MSI-H disease, moderately infiltrated with TILs. Duration of response was 7.3 and 8.1+ months, respectively. Authors reported a median PFS of 1.7 months and a median OS of 9.6 months. Drug related severe adverse events (colitis and rash) occurred only in two patients, no G4-5 related AEs were reported [80] A Phase 2 study with an anti-PD-L1 antibody was recently presented at ASCO 2017: it is an open-label, two stage trial in which Avelumab 10 mg/kg was administered biweekly to women with recurrent or persistent endometrial cancer. Patients were divided in two cohorts on the basis of the MMR proteins expression. In the first stage, 16 patients will be enrolled in each cohort, if at least two objective responses or two PFS at six months were observed accrual will continue to the second stage. The trial is ongoing and until now 16 patients have been enrolled: 13 in the MSS cohort and 3 in the MSI/POLE cohort. Co-primary endpoints are ORR and rate of PFS at six months [81]. The results of the preliminary clinical data are summarized in Table 1.

**Table 1:** Published and preliminary data of trials evaluating the activity of checkpoint inhibitors in the treatment of endometrial cancer

Kind of treatment	Number of patients	Phase	Class of experimental agent	Line of therapy	ORR	PFS	OS	Study name/First author
Pembrolizumab 10 mg/kg every 2 weeks	24	Ib	Anti-PD-1	2L+	13%	19% at six months	68.8% at six months	KEYNOTE-028
Lenvatinib 20 mg/day + Pembrolizumab 200 mg every 3 weeks	23	Ib/II	Multikinase inhibitor + Anti-PD-1	2L+	48%	Not estimable	Not estimable	Vicky Makker
Atezolizumab 1200 mg or 15 mg/kg IV q3w	15	Ia	Anti-PD-L1	2L+	13%	1.7 months	9.6 months	Gini F. Fleming

PFS = Progression Free Survival; ORR = Overall Response Rate; OS = Overall Survival.

As discussed above another possible target for checkpoint inhibitors is CTLA-4, in order to prevent binding with its ligand B7. Ipilimumab and Tremelimumab are monoclonal antibodies able to disrupt this interaction. This approach has proven to be effective in the treatment of melanoma [82, 83], but data supporting the effectiveness of anti-CTLA4 in the treatment of EC have not been reported so far.

## DISCUSSION AND FUTURE PERSPECTIVES

The use of checkpoint inhibitors has a strong rationale in EC, however clinical development is at very beginning and, despite preliminary encouraging results, several issues need to be addressed.

First of all, few data are available regarding predictive biomarkers of response to checkpoint inhibitors. In order to select patients who mostly benefit from these therapies more and more studies analyze cancer genome and its correlation with TME. PD-L1 expression level has been reported as a predictive biomarker of response in NSCLC [63, 65] but its predictive role is not consistent across different cancers types. This may be related to various detection strategies and different specimens analyzed (before, during or after treatments) [66]. Moreover, a crucial role in immune surveillance is played by the others cells expressing PD-L1 present in TME, as reported in a translational study by Webb and colleagues for tumor-associated macrophages (TAMs) on the basis of tissue microarrays of optimally debulked ovarian cancers [84].

Recently, huge progress has been achieved regarding the relationship between cancer genome and response to checkpoint inhibitors [85, 86]. A relationship between response to PD-1/PDL-1 inhibitors and somatic mutations load has been reported in melanoma and lung cancer [67, 68] according to the hypothesis that identifying neo-antigens generated by mutations is an essential step for immune response. Indeed, mutational burden defines immunogenicity of cancers [87, 88]. Besides the neo-antigens loads, some studies were conducted in the melanoma to identify mechanisms related to resistance to PD-1 inhibitors. Shin and colleagues found that loss of function mutations in JAK1 and JAK2, where associated with a deficiency of interferons that physiologically induce PD-L1 upregulation, in several melanoma cells lines which correlate to resistance to checkpoints inhibitors. Moreover, they reported beta-2 microglobulin deletions or mutations leading to beta-2 microglobulin inactivation and subsequent inability for T cells to recognize the tumor. These mutations are responsible for primary resistance or can be developed during treatment, arising secondary resistance to checkpoints inhibitors [89, 90]. The importance of the interferon associated pathways for the response to anti PD-1 and CTLA-4 was confirmed also by an MD Anderson report [91].

As reported above, two subgroups of EC, POLE-ultra-mutated and MSI-H, are characterized by higher number of neo-antigens and the elevated amount of TILs [15]. The consequent high immunogenicity of POLE-ultramutated EC is speculated to be responsible for the good prognosis and possibly for likelihood of responding to immune checkpoint inhibitors [92]. Neo-antigen load could possibly be a biomarker of response also in hypomutated EC, as reported by Shukla and colleagues [93]. Indeed they observed that hypo-mutated tumors with highest neo-antigen load have a better PFS. Moreover, they reported that a lower neo-antigens number is associated with particular gene alterations, CTNNB1 and PIK3CA mutations and MYC amplifications. The above variations could be used as indicators of less immunogenicity and, consequently, of lower response rate to checkpoint inhibitors.These discoveries could help clinicians to identify EC patients that could benefit from checkpoint inhibitors.

The need to identify possible biomarkers of response is also crucial for the ongoing clinical trials, reported in Table 2, which, following evidence derived from other cancers, are exploring combinations of checkpoints inhibitors or associations of checkpoints inhibitors with chemotherapy, small tyrosine kinase inhibitors (TKIs) and mTOR inhibitors.

**Table 2:** Ongoing trialsusing checkpoint inhibitors in endometrial cancer

Combination	Treatment setting	Line of therapy	Phase	Primaryendpoint	Status	Trial identifer
aPD-L1	Avelumab in Patients With MSS, MSI-H and POLE-mutated	2L+	2	PFS6	Recruiting	NCT02912572
aPD-1	Pembrolizumab in Ultramutated and Hypermutated EC	2L+	2	ORR, safety by CTCAE v4	Recruiting	NCT02899793
aPD-1	Pembrolizumab on the TumoralImmunoprofile of Gynecologic Cancers	1L	1	Tumor immune infiltrates	Recruiting	NCT02728830
aPD-1	MK-3475 Immunotherapy in Endometrial Carcinoma	1L	1	Safety by CTCAE v4	Recruiting	NCT02630823
aPD-1 + Chemo	Pembro/Carbo/Taxol	1L+	2	ORR	Not yet recruiting	NCT02549209
aPD-1 + Bev/C/PLD	IMGN853 + Bevacizumab, Carboplatin, PLD or Pembrolizumab	2L+	1	ORR, SAEs, TEAEs	Recruiting	NCT02606305
aPD-1 + TKI	Pembrolizumab + Lenvatinib	2L+	1b/2	MTD, DLTs, ORR	Recruiting	NCT02501096
aPD-1 + TIL	Pembrolizubab+ TIL PBL and aldesleukin	2L+	2	ORR	Recruiting	NCT01174121
aPD-1 + TKI	Pembrolizumab +Itacitinib	2L+	1	Safety by CTCAE v4	Recruiting	NCT02646748
aPD-L1 + aCTLA-4	Durvalumab +/− Tremelimumab	2L+	2	ORR	Recruiting	NCT03015129
aPD-1 + Chemo	Nivolumab + Chemotherapy	2L+	1b/2	RP2D	Recruiting	NCT02423954
aPD-1 + aCTLA-4	Nivolumab + Ipilimumab	2L+	2	ORR	Not yet recruiting	NCT02982486
aPD-1 + mTORi	Nivolumab + Temsirolimus/ Nivolumab + CT	2L+	1b/2	RP2D	Recruiting	NCT02423954
aPD-1 + aCTLA-4	Nivolumab + Ipilimumab in rare tumors	1L+	2	ORR	Recruiting	NCT02834013
aPD-L1 + Chemo	Atezolizumab + Carboplatin-cyclophosphamide	2L	1	Toxicity by CTCAE v4	Recruiting	NCT02914470
aPD-L1 + IDO Inhibithor	Atezolizumab + GDC-0919	2L+	1	DLT, SAEs	Recruiting	NCT02471846

Chemo, chemotherapy; Bev, bevacizumab; C, Carboplatin; PLD, pegylatedliposomaldoxorubicin; TKI, tyrosine-kinaseinhibitor; TIL, tumorinfiltratinglymphocytes; mTORi, mTORinhibitor; IDO, indoleamine 2,3-dioxigenase; L, line (regime of chemotherapy); PFS6, progression free survivalat 6 months;ORR, overallresponse rate; CTCAE v4, Common TerminologyCriteria for AdverseEvents, version 4.03; SAEs, seriousadverseevents; TEAEs, treatment-emergentadverseevents; MTD,maximum tolerated dose; DLT, dose-limitingtoxicities; RP2D, recommendedphase 2 dose; NCT, National Clinical Trial.

The study published by Pakish evaluating the immune infiltrate in MSI-H EC suggests for the first time that among patients with MSI, there may be differences in the TME on the basis of hereditary or sporadic nature of the MMR deficiency [4]. These data have been recently confirmed at the Society for Gynecologic Oncology Annual Meeting on Women's Cancer by Ring and colleagues, who reported that EC related to LS has a stronger expression of PD-L1, in particular for LS caused by *MSH6* loss [94].

For this reason, future clinical trials should stratify patients considering Lynch related and sporadic MSI-H tumor.

More recently, the European Society of Medical Oncology (ESMO) provided a practical guidance for MMR-deficiency testing in EC [95] underlying its emerging importance both to guide adjuvant treatment and to identify LS cases. Although there is not a general agreement on testing EC patients for LS, the above data suggest that MMR-deficiency could be predictive of response to immunotherapy and help clinicians in their therapeutic choices. At present, both MSI (pentaplex panel) and IHC are validated methods in EC testing [96, 97].

### Clinical settings and associations

Considering its important role in EC, one of the most promising partners of checkpoint inhibitors is radiotherapy. In particular, as suggested for other malignancies, the so called out-of-the-field (abscopal) responses in patients receiving radiation therapy during immunotherapy may be relevant also in EC [98]. Another important direction of clinical research is represented by the association of chemotherapy and immune checkpoint inhibitors [99]. Although chemotherapies are believed to be immunosuppressive, when given at the right dose and sequence may provide a “priming” effect for the immune system. Trials have shown already that platinum based chemotherapy associated with immune checkpoint inhibitors is active in NSCLC [100]. Since chemotherapy (especially platinum based chemotherapy) is also active in EC, it is likely to obtain similar results with the addition of a checkpoint inhibitor.

Considering other malignancies where the development of immune checkpoint inhibitors is more advanced (e.g. melanoma), there is no clear evidence suggesting a significant improvement in survival [69]. Accordingly, it is therefore more likely that patients with advanced endometrial cancer (stage III and IV) may benefit best from immune checkpoint inhibitors.

## CONCLUSIONS

ECs have already proved to be an immunogenic diseases suggesting a potential role for checkpoints inhibitors in their treatment. As reviewed above, the TCGA classification is a step forward towards individualized therapies and should be considered in future clinical trials, to assess which subsets of EC patients are more likely to benefit from an immunotherapeutic approach. Further investigations should include the identification of which dominant immunosuppressive pathway characterizes each subtype in order to better identify reliable biomarkers of response. Future strategies will explore different clinical settings and combinations of chemo and radiotherapy with checkpoint inhibitors to boost immune response and improve patients outcomes.

## MATERIALS AND METHODS

### Search strategy

We conducted a search on Medline with Mesh keywords: endometrial cancer, endometrial carcinoma, endometrial neoplasm, endometrium cancer, endometrium carcinoma, and endometrium neoplasm. Moreover, the search strategy included terms for endometrial cancer matched with immunotherapy; tumor infiltrating lymphocytes (TILs); polymerase epsilon (POLE)-ultra-mutated; microsatellite instability (MSI); tumor-microenvironment; programmed death-1 (PD-1); programmed death-ligand 1 (PD-L1);cytotoxic T-lymphocyte-associated protein 4 (CTLA-4); the name of all checkpoint inhibitors discussed in the paper. The literature search was performed up to June 2017. Moreover had searched abstract books of conference proceedings between 2010 and 2017 to identify potentially eligible studies. With the same keywords we operated a search on clinicaltrials.gov.

### Selection criteria

Retrevied articles were examined by all coauthors to assess their consistency with the aims of the article.

### Abbreviations

Not applicable.
